# Genome-Wide Association Study of Body Weight Traits in Chinese Fine-Wool Sheep

**DOI:** 10.3390/ani10010170

**Published:** 2020-01-19

**Authors:** Zengkui Lu, Yaojing Yue, Chao Yuan, Jianbin Liu, Zhiqiang Chen, Chune Niu, Xiaoping Sun, Shaohua Zhu, Hongchang Zhao, Tingting Guo, Bohui Yang

**Affiliations:** 1 Lanzhou Institute of Husbandry and Pharmaceutical Sciences, Chinese Academy of Agricultural Sciences, Lanzhou 730050, China; luzengkui@caas.cn (Z.L.); yueyaojing@126.com (Y.Y.); yuanchao@caas.cn (C.Y.); liujianbin@caas.cn (J.L.); niuchune@caas.cn (C.N.); sunxiaoping@caas.cn (X.S.); zhu87932890@outlook.com (S.Z.); 18837101296@163.com (H.Z.); 2 Sheep Breeding Engineering Technology Research Center of Chinese Academy of Agricultural Sciences, Lanzhou 730050, China; 3 Novogene Bioinformatics Institute, Beijing 100029, China; chenzhiqiang@novogene.com

**Keywords:** body weight, genome-wide association study, fine-wool sheep, genomic analysis, functional analysis

## Abstract

**Simple Summary:**

Body weight traits are economically important in the sheep industry, and it is critical to explore their underlying genetic architecture. Hence, four body weight traits, including birth, weaning, yearling, and adult weights were examined. Through a genome-wide association study on Chinese fine-wool sheep, several candidate single-nucleotide polymorphisms (SNPs) and genes were found potentially associated with the traits of interest. The results of this study may facilitate the potential use of the genes involved in growth and production traits for the genetic improvement of productivity in sheep.

**Abstract:**

Body weight is an important economic trait for sheep and it is vital for their successful production and breeding. Therefore, identifying the genomic regions and biological pathways that contribute to understanding variability in body weight traits is significant for selection purposes. In this study, the genome-wide associations of birth, weaning, yearling, and adult weights of 460 fine-wool sheep were determined using resequencing technology. The results showed that 113 single nucleotide polymorphisms (SNPs) reached the genome-wide significance levels for the four body weight traits and 30 genes were annotated effectively, including *AADACL3*, *VGF*, *NPC1*, and *SERPINA12*. The genes annotated by these SNPs significantly enriched 78 gene ontology terms and 25 signaling pathways, and were found to mainly participate in skeletal muscle development and lipid metabolism. These genes can be used as candidate genes for body weight in sheep, and provide useful information for the production and genomic selection of Chinese fine-wool sheep.

## 1. Introduction

Genome-wide association studies (GWAS) are used to screen the whole genome for target genes that correlate with phenotypic traits, using single nucleotide polymorphisms (SNPs) as genetic markers. They have become an important method for identifying candidate genes for important economic traits in livestock. Compared with traditional quantitative trait locus (QTL) mapping, GWAS have a greater capability to detect causal mutations, and can detect mutations in a smaller genetic range [1]. In recent years, GWAS have been included in genetic breeding programs for animals like pigs, cattle, sheep, and chickens, and have identified many genes or molecular markers that could regulate important economic traits in livestock [2,3,4,5].

Body weight is the most important growth and development index in sheep production, and it influences meat and wool production, and reproduction of sheep, both directly and indirectly [6]. During production, the birth, weaning, yearling, and adult weights are used to reflect the growth and development of sheep. The growth in body weight is influenced by a combination of the genetic background of the sheep and its feeding and management. Growth in weight is a highly heritable trait and is one of the main indexes of selection [6]. Ghasemi et al. carried out a GWAS on the birth weight of Lori-Bakhtiari sheep and identified three genes (*RAB6B*, Tf serotransferrin and *GIGYF2*) on chromosome one as candidate genes for this trait [4]. In addition, Al-Mamun et al. performed a GWAS on the weaning weight of Australian Merino sheep and identified three genes (*LAP3*, *NCAPG,* and *LCORL*) on chromosome six for use during marker-assisted selection (MAS) for body weight [7]. Other genome scanning and QTL mapping studies of body weight in sheep also found many candidate genes that significantly correlated with body weight traits [8,9,10,11]. However, while studies mapping candidate genes that are related to body weight traits in sheep have been performed, the major genes are still unknown. Additionally, many previous studies only examined the candidate genes that significantly correlated with body weight during a single developmental stage, rather than performing a systematic study of body weight traits throughout the different developmental stages.

In this study, the genome-wide associations of the birth, weaning, yearling and adult weights of 460 fine-wool sheep in China were determined using genome resequencing technology. SNPs and candidate genes, related to body weight, were identified, thereby, providing references for the MAS of body weight in fine-wool sheep.

## 2. Materials and Methods

### 2.1. Animals and Sample Collection

All experimental protocols and procedures were approved by the Institutional Animal Care and Use Committee of Lanzhou Institute of Husbandry and Pharmaceutical Science of Chinese Academy of Agricultural Sciences (Approval No. NKMYD201805; Approval Date: 18 October 2018). A total of 460 fine-wool sheep in China were selected for this study, including 220 Alpine Merino sheep (AMS; 75 males and 145 females; Huangcheng, Gansu, China), 120 Chinese Merino sheep (CMS; 60 males and 60 females; Gongnaisi, Xinjiang, China), 60 Aohan fine-wool sheep (AHS; 30 males and 30 females; Chifeng, Inner Mongolia, China), and 60 Qinghai fine-wool sheep (QHS; 30 males and 30 females; Sanjiaocheng, Gangcha, China). All the sheep were selected randomly, without pedigree information. The sheep were farmed on pasture, with appropriate feed being supplemented in winter. Blood samples (5 ml) were collected from the neck veins of all the sheep and placed in EDTA anti-freezing tubes for storage at −20 °C.

### 2.2. Phenotypic Measurements

The birth, weaning (3.5 months), yearling (12 months), and adult (30 months) weights of all sheep were measured using an electronic scale, and gender was also recorded. Birth weights were recorded within 0.5 h after birth, and the weaning, yearling, and adult weights were measured after 12 h of fasting. Phenotypic values that aligned absolutely or approximately to a normal distribution were not processed, while extremely deviated values underwent Box-Cox transformation using the powerTransform and bcPower functions in the car extension package in R, in order to obtain an approximately normal distribution [12,13].

### 2.3. DNA Resequencing and Data Preprocessing

Genomic DNA was extracted from each blood sample using the standard phenol-chloroform method [14]. The integrity and purity of the DNA were tested using 1% agarose gel electrophoresis and a Nanodrop 2000 ultraviolet spectrophotometer (Thermo, Waltham, MA, USA). The DNA concentration was measured using a Qubit 2.0 (Invitrogen, Carlsbad, CA, USA). Aliquots (1.5 µL) of DNA were taken from each sample and library construction was performed according to the Truseq Nano DNA HT (Illumina, San Diego, CA, USA) instructions. Based on this library, the whole genomes were re-sequenced with paired-end 150 bp reads using the Illumina HiSeq Xten platform (Illumina), producing the raw data. Index sequences, barcoding sequences, and low-quality sequences were removed by Trimmomatic software (v0.32), with the following parameters being used: MINLEN = 50, LEADING = 20, TRAILING = 20, SLIDINGWINDOW = 5, 20 [15]. The clean reads were compared with the sheep reference genome (Oar_v4.0, GCF_000298735.2) using BWA software (v0.7.11), with the following alignment parameters being set: mem -t 4 -k 32 -M [16]. Duplicates were removed using the rmdup command in SAMTools [17]. If multiple read pairs had identical external coordinates, only the pair with the highest mapping quality was retained.

### 2.4. SNP Identification and Annotation

High-quality SNPs were obtained through SNP detection using the mpileup command in SAMTools, with the filtering conditions set as follows: Coverage depth > 3, proportion of mis-assignments < 10%, and minor allele frequency > 5% [17]. The high-quality SNPs obtained using these steps were annotated using ANNOVAR software [18]. The annotation analysis mainly included the genes within which the SNP loci were located, and the variations in the types and positions of the SNP loci.

### 2.5. Genome-wide Association Studies

The correlations between the SNPs and the traits were tested using mixed linear models in GEMMA software [19,20]. In GWAS, individual kinship and population stratification are the main causes of false positive correlations. Therefore, population genetic structure and sex were used as a fixed effect, and individual kinship was used as a random effect to correct for the influences of population structure and individual kinship. The statistical analysis model used in this study was y = Xα + Zβ + Wμ + e, where y is the phenotypic trait, X is a matrix of fixed effects, α is the estimation parameter of the fixed effects, Z is a matrix of SNPs, β is the effect of the SNPs, W is a matrix of random effects, μ is the predicted random individuals, and e is the random error, with the distribution e~ (0, δ_e_^2^). The significance threshold for the GWAS was defined using the Bonferroni correction method. The total type I error rate was controlled at 5% and the significance threshold of the genome was 0.05/Nsnp, where Nsnp is the number of SNPs remaining after quality control [21].

### 2.6. Bioinformatics Analysis

Genes associated with significantly correlated SNP loci were annotated with the sheep reference genome (Oar_v4.0, GCF_000298735.2) and submitted to the DAVID database (http://david.abcc.ncifcrf.gov/) for gene ontology (GO) and Kyoto encyclopedia of genes and genomes (KEGG) analysis [22,23]. Significant enrichment in the candidate genes was indicated by a *p*-value of ≤0.05.

### 2.7. Statistical Analysis

All body weight data reported are expressed as mean ± SD. Student’s t-test was carried out using SPSS software for statistical analysis of the data. A *p* value of <0.05 was considered to be statistically significant.

## 3. Results

### 3.1. Phenotypic Data Analysis of Body Weight Traits

The weight traits of the 460 fine-wool sheep of four breeds measured in this study included the birth, weaning, yearling, and adult weights. The descriptive statistics calculated for the body weight traits, included the mean, median, maximum, and minimum values, as well as the standard deviation. It can be seen from Supplementary Figure S1 that the rams were heavier than the ewes, and that the AHS and AMS were heavier than the CMS and QHS, during all phases of growth. Additionally, a frequency distribution chart of the phenotypic values for these traits was drawn, and an approximately normal distribution was attained after the adjustment of any values that did not align to the normal distribution. 

### 3.2. Summary of Sequencing Data

After sequencing was performed using the Illumina HiSeq Xten platform, high-quality next-generation sequencing data for the 460 fine-wool sheep were obtained (Supplementary Table S1). A total of 8,222,158,631,700 raw bases were generated in this study, with 8,189,580,053,700 clean bases being retained after quality control, at an average retention rate of 99.60%. The GC contents of the 460 samples ranged from 41.58% to 47.31%, thus, conforming to the base composition laws. Q20 ≥93.75% and Q30 ≥85.83%. Over 97.44% of the clean reads could be aligned to the sheep reference genome using BWA software. The coverage depth of each sample after genome alignment was about 8.59 times. Following quality control, 12,533,461 high-quality SNPs were obtained.

### 3.3. Genome-Wide Association Study

Before performing the GWAS analysis, the population structure of the test population had to be analyzed and corrected accordingly [24]. As can be seen from Supplementary Figure S2, with the exception of QHS, the other three breeds are well-separated. Therefore, the effect of population stratification on phenotypic variation needs to be corrected when performing association analysis. The linkage disequilibrium (LD) analysis was performed on the four breeds and the LD decay is shown in Supplementary Figure S2. The results indicate that LD decay tends to be stable when the distance is 100 kb. Therefore, genes located within 100 kb of the significant SNPs are defined as candidate genes.

**Figure 1:**
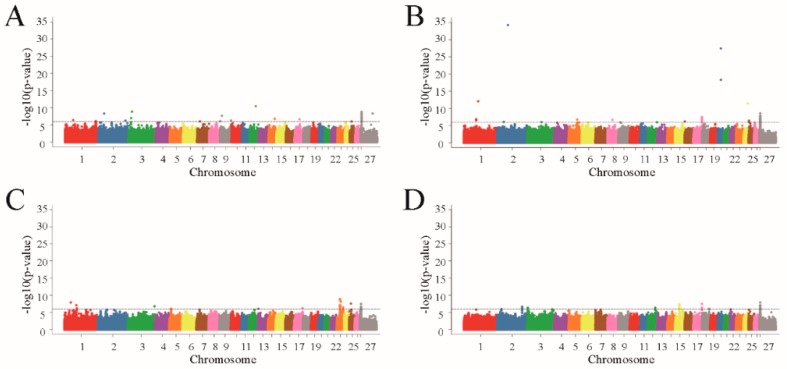
Manhattan plots of body weight traits for four fine-wool sheep breeds. The grey horizontal lines in the Manhattan plots indicate the suggestive significance (10^−6^) thresholds. (**A**) birth weight; (**B**) weaning weight; (**C**) yearling weight; (**D**) adult weight.

GEMMA software was used to perform whole-genome correlation analysis based on a mixed linear model for the birth, weaning, yearling, and adult weights. The GWAS results showed that a total of 113 SNPs in the genome correlated significantly with the four body weight traits (Figure 1).

**Figure 2:**
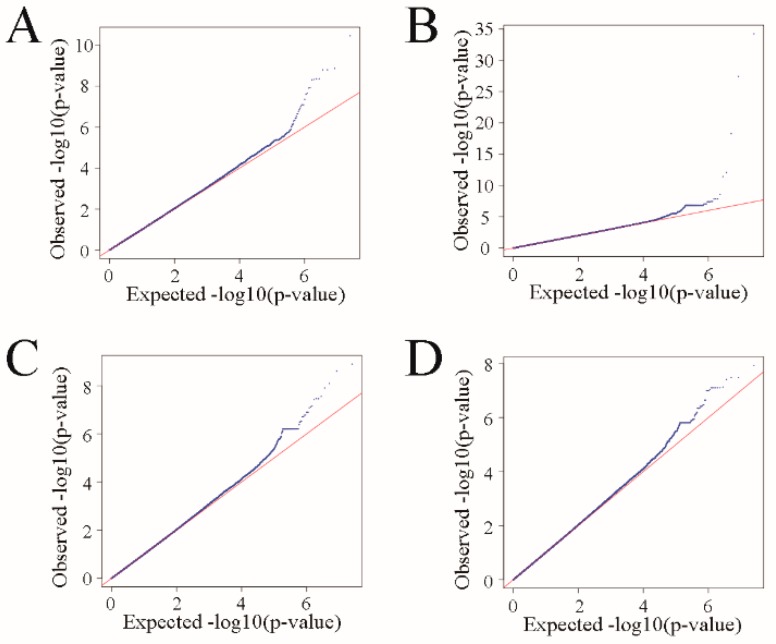
Quantile-quantile plots of body weight traits using mixed linear model approach. Blue dots represent the −log10 (*p*-value) of the entire study and the red line represents the expected values under the null hypothesis of no association. (**A**) birth weight; (**B**) weaning weight; (**C**) yearling weight; (**D**) adult weight.

For birth weight, 29 significantly correlated SNPs were detected on the chromosomes Chr1, Chr2, Chr3, Chr7, Chr9, Chr12, Chr14, Chr17, Chr25, and Chr27, and were effectively annotated to eight genes (Figure 1A; Table 1). For weaning weight, 38 significantly correlated SNPs were detected on Chr1, Chr2, Chr3, Chr5, Chr8, Chr16, Chr17, Chr20, Chr24, Chr25, and Chr27, and effectively annotated to 10 genes (Figure 1B; Table 1). For yearling weight, 31 significantly correlated SNPs were detected on Chr1, Chr3, Chr5, Chr13, Chr17, Chr23, Chr25, and Chr27, and effectively annotated to 12 genes (Figure 1C; Table 1). For adult weight, 15 significantly correlated SNPs were detected on Chr2, Chr3, Chr12, Chr15, Chr17, Chr18, and Chr27, and effectively annotated to six genes (Figure 1D; Table 1). As can be seen on the Q-Q chart, the observed and expected *p*-values for the four traits were clearly separated by 10^−4^, indicating that the correlation results were good (Figure 2).

### 3.4. Bioinformatic Analysis

**Figure 3:**
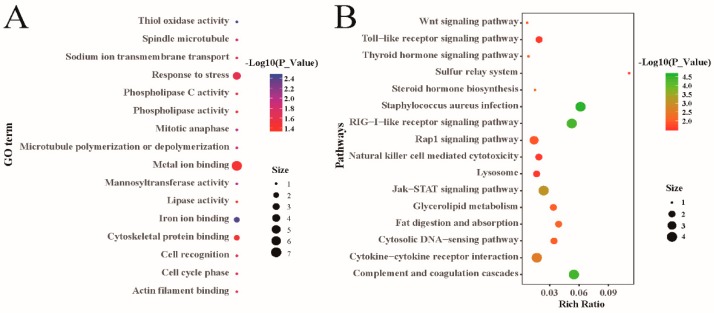
Gene ontology (GO) (**A**) and Kyoto encyclopedia of genes and genomes (KEGG) (**B**) analysis for the regional candidate genes with genome-wide significant association.

To further understand the functions of the significantly-correlated SNPs, functional enrichment analysis of the genes to which the SNPs were annotated was carried out. A total of 78 GO terms were significantly enriched (*p* < 0.05), including 25 biological processes, 13 cellular components, and 40 molecular functions (Supplementary Table S2). These GO terms were mainly related to microtubular movement, the cytoskeleton and the stress response, among others (Figure 3A). The KEGG pathway analysis revealed that the genes associated with the significantly correlated SNPs were highly enriched in 25 pathways (*p* < 0.05, Supplementary Table S3). Further analysis indicated that these pathways, which included the toll-like receptor signaling pathway, the wnt signaling pathway, and fat digestion, were associated with immunological functions, the development of skeletal muscle, and lipid metabolism (Figure 3B).

## 4. Discussion

Body weight is an important economic trait in sheep production. Studies have shown that the heritability of sheep body weight is moderately high, with the heritability of birth weight and weaning weight ranging from 0.30 to 0.35, the heritability of yearling weight being 0.40–0.45, and that of adult weight being 0.39 [25]. A trait with a higher heritability can be more accurately selected. It is, therefore, vitally important for sheep production and breeding that the complex molecular mechanisms behind sheep body weight and the important functional genes that influence this trait are identified.

Population stratification is an important factor causing false positive GWAS results [26]. Studies have shown that using a mixed linear model that simulates population structure, kinship, and family structure is the most effective method of reducing the effects of population stratification currently available [24]. Therefore, in this study, a population genetic structure was included as a fixed effect and individual kinship as a random effect, to correct for the effects of population structure and individual kinship relationships. It can be seen from the Q-Q plots of the four body weight traits that no population stratification phenomena were apparent, based on the observed and expected *p*-values. However, this phenomenon was apparent for the SNPs, which were highly correlated with the body weight traits. The *p*-values of the significantly correlated SNPs in this study were smaller than 10^-6^, so it is probable that these SNP correlations do exist.

The genome-wide association analysis of the birth, weaning, yearling, and adult weights of 460 fine-wool sheep was performed using a mixed linear model in this study, and a total of 113 SNPs reached genome-wide significance after detection. Interestingly, except for sex chromosomes, significantly correlated genes on autosomes in the four body weight traits were all different. We, therefore, speculated that the sheep body weight was controlled by different genes during different phases, and that the body weight within a single phase was regulated by many genes. Sheep body weight is a quantitative trait that is influenced by both the genotype and the environment. The polygene hypothesis for quantitative traits, therefore, supports this speculation that body weight is regulated by micro-effect genes. It has also been found in a GWAS study of live weights in chickens that weight traits are regulated by micro-effect genes [27]. Gene expression is selective and affected by time, space, and environmental factors. Except for a very small number of genes that maintain stable expression in any external environment (e.g., *β-actin* genes), whether most genes are expressed and the level of expression, are regulated based on changes in the external environment. Environmental factors, include material aspects, such as nutritional status. However, psychological factors, such as stress are also relevant. These environmental factors can change the endocrine system of the body, which in turn changes the expression of genes. The fine-wool sheep that we chose are mainly grazing animals and the nutritional level of their pastures varies among the seasons, which affects their gene expression. It has also been found in other studies that different levels of feed nutrition can alter gene expression [28,29]. Secondly, grazing sheep, stimulated by hot summers and cold winters, can also change gene expression [30,31].

In addition, we found that the candidate body weight genes, identified in this study, had poor repeatability, compared with previously reported GWAS results for sheep body weight [4,7,10,32]. In previous sheep body weight GWAS, variation detection was performed using BeadChips, so only a very limited number of mutations were detected. The number of mutations that can be detected using the resequencing technique, used in this study, is several orders of magnitude higher than can be identified using BeadChips. Furthermore, the four sheep breeds selected for this study were established by long-term artificial selection and the linkage disequilibrium level of their genomes was thus very high. As a result, some SNPs unrelated to the measured traits displayed rising signal values, caused by linkage with target SNPs, which may have influenced the signal values of the target SNPs.

Body weight growth is closely related to the growth of muscle, fat, and bone tissues; candidate genes that correlated with the growth and development of these tissues were identified in this study. The *AADACL3* (arylacetamide deacetylase like 3) gene located on chromosome 12 that correlated with birth weight participates in fat metabolism [33]. The *VGF* (nerve growth factor inducible) gene on chromosome 24, which correlates with weaning weight, is involved in regulating the food intake and body weight of animals, with the knockout of the *VGF* gene in mice leading to weight reduction, in vivo fat reduction, and excessive energy consumption [34,35,36,37]. The *VGF* gene may also regulate fat synthesis and decomposition [38,39]. The *NPC1* (niemann-Pick Type C) gene on chromosome 23 that correlated with yearling weight participates in controlling the steady state of animal energy metabolism and plays an important regulatory role in body weight and fat metabolism [40,41,42]. The *SERPINA12* (serpin family A member 12) gene on chromosome 18 that correlated with adult weight is closely associated with animal body composition and lipid distribution, and could serve as a marker for lipid metabolism [43,44].

Many of the genes that were found to be associated with significantly correlated SNPs in this study were enriched in GO terms, such as actin filament binding (*p* < 0.05). The long-term and complex process of animal muscle development mainly relies on the proliferation and hypertrophy of muscle fibers [45]. Actin plays a vital role in the transformation of myotubes into myofibers [46]. Many of the other significantly-enriched GO terms, such as microtubule and cytoskeletal protein, are also essential for muscle development.

The KEGG pathway analysis revealed that the wnt signaling pathway was remarkably enriched for the adult weight trait. Belonging to the secretory glycoprotein family, wnt participates in the proliferation and differentiation of multiple precursor cells [47]. Previous research has found that the wnt signaling pathway is indispensable for embryonic and postnatal skeletal muscle homeostasis [48]. During embryonic skeletal muscle development, the wnt signaling pathway induces myogenesis, mainly by regulating *MRFs* (myogenic regulatory factors) [49,50]. During skeletal muscle development, after birth, the classical wnt signaling pathway mainly regulates the differentiation of skeletal muscle satellite cells, while the nonclassical wnt signaling pathway mainly mediates the self-renewal of skeletal muscle satellite cells and the growth of muscle fibers [51,52]. Bone growth and development are closely related to body weight, with bone development preceding skeletal muscle development, and the wnt signaling pathway also exerts an important effect on the bone development process [53,54]. However, in addition to the wnt signaling pathway, the role of the Jak-STAT (janus kinase- signal transducer and activator of transcription) signaling pathway in satellite cells has also gradually been revealed in recent years. The Jak1/STAT1/STAT3 pathway promotes the proliferation of activated satellite cells and prevents their premature differentiation into myotubes. The Jak2/STAT2/STAT3 pathway also mediates the positive regulation of satellite cell differentiation by *MyoD* (myoblast determination protein) and *MEF2* (myocyte enhancer factor 2) [55,56]. Some GO terms related to lipid metabolism were also enriched and these GO terms were also impacted by the environment in which the fine-wool sheep selected for this study were located. The four fine-wool breeds selected for this study were mainly grazed in areas that had severe fodder grass shortages during spring and winter. As they lived in a harsh environment, lipid metabolism was critically important for their production and reproduction. Furthermore, this hostile environment also impacted the sheep’s health, and we found that the significantly enriched pathways included some signaling pathways related to body immunity, such as the toll-like receptor signaling pathway.

## 5. Conclusions

In conclusion, this study identified genomic regions, and gene-associated biological pathways, related to the body weight traits of Chinese fine-wool sheep. The identified candidate genes were found to mainly participate in skeletal muscle development and lipid metabolism. The genes identified are good candidates for further functional validation to uncover the biological mechanisms underlying body weight variation in Chinese fine-wool sheep.

## Figures and Tables

**Table 1:** Genome-wide association studies (GWAS)-identified significant single-nucleotide polymorphisms (SNPs), associated traits, and nearest candidate genes.

Traits	Chr	Position (bp)	MAF	−log10 (*p*-Value)	Distance (bp)	Gene	Variant Effect
birth weight	ch1	253212365	0.10	6.111011551	−19101	*SLCO2A1*	intergenic
	ch9	14382214	0.49	7.664224268	−2084	*LY6K*	intergenic
	ch9	90030866	0.15	6.323004957	−26215	*RALYL*	intergenic
	ch12	52839429	0.50	10.45675318	0	*AADACL3*	intronic
	ch17	39807635	0.06	6.65587487	0	*C17H4orf45*	intronic
	ch25	13791395	0.48	6.006388368	−17811	*BICC1*	intergenic
	ch27	6893599	0.16	8.797304666	10577	*GPR143*	intergenic
	ch27	7049963	0.15	8.355957873	0	*SHROOM2*	intronic
weaning weight	ch1	101447761	0.21	6.814810126	7370	*C1H1orf68*	intergenic
	ch1	118783826	0.50	12.06478576	−16239	*CLIC6*	intergenic
	ch3	118519704	0.07	6.007008521	0	*TMTC2*	intronic
	ch20	26410829	0.50	27.43388012	−16968	*STK19*	intergenic
	ch20	26410829	0.50	27.43388012	−25619	*DXO*	intergenic
	ch20	26410829	0.50	27.43388012	−27940	*SKIV2L*	intergenic
	ch24	35415932	0.49	11.41651058	−19192	*NAT16*	intergenic
	ch24	35415932	0.49	11.41651058	−28975	*VGF*	intergenic
	ch27	6892015	0.21	8.574556919	8993	*GPR143*	intergenic
	ch27	6892015	0.21	8.574556919	−87231	*SHROOM2*	intergenic
yearling weight	ch1	51280492	0.50	7.90882916	−29110	*RABGGTB*	intergenic
	ch1	96929132	0.50	7.121270413	29522	*TRNAQ-CUG*	intronic
	ch1	96929132	0.50	7.121270413	27565	*TRNAN-GUU*	intronic
	ch1	101447761	0.21	6.206583675	7370	*C1H1orf68*	intergenic
	ch5	13636592	0.11	6.080914687	17967	*ZNF557*	intergenic
	ch13	1884594	0.35	6.049430267	0	*PLCB4*	intronic
	ch23	33324859	0.50	8.093591468	0	*NPC1*	intronic
	ch23	33324859	0.50	8.093591468	−16422	*C23H18orf8*	intronic
	ch23	52260021	0.12	6.009205597	0	*DCC*	intronic
	ch25	12802212	0.50	7.554054255	−22550	*ZNF25*	intergenic
	ch27	7053187	0.19	7.464649666	170165	*GPR143*	intronic
	ch27	7053187	0.19	7.464649666	0	*SHROOM2*	intronic
adult weight	ch2	206466290	0.06	6.642091631	0	*PARD3B*	intronic
	ch3	4796131	0.09	6.34382137	0	*MED27*	intronic
	ch18	57956306	0.11	6.049410169	24508	*SERPINA14*	intergenic
	ch18	57956306	0.11	6.049410169	−4231	*SERPINA12*	intergenic
	ch27	6743376	0.05	7.929222061	0	*TBL1X*	intronic
	ch27	6997506	0.19	6.124895773	0	*SHROOM2*	intronic

Chr: chromosome; MAF: minor allele frequency; Variant effect: SNP effect on the gene.

## Supplementary Materials

The following are available online at https://www.mdpi.com/2076-2615/10/1/170/s1, Figure S1: Violin plots showing distribution of the body weight in each group; Figure S2: Population structure and distribution of LD for four fine-wool sheep breeds; Table S1: Sequencing reads, alignment statistics and mean genome-wide coverage of each sample. Table S2: GO analysis for the regional candidate genes with genome-wide significant association. Table S3: KEGG analysis for the regional candidate genes with genome-wide significant association.
