# Supplementary figures and images for: Genome-Wide Association Study of Body Weight Traits in Chinese Fine-Wool Sheep
Source: Animals (Basel). 2020 Jan 19;10(1):170. doi: 10.3390/ani10010170 (PMC7022301; doi:10.3390/ani10010170)

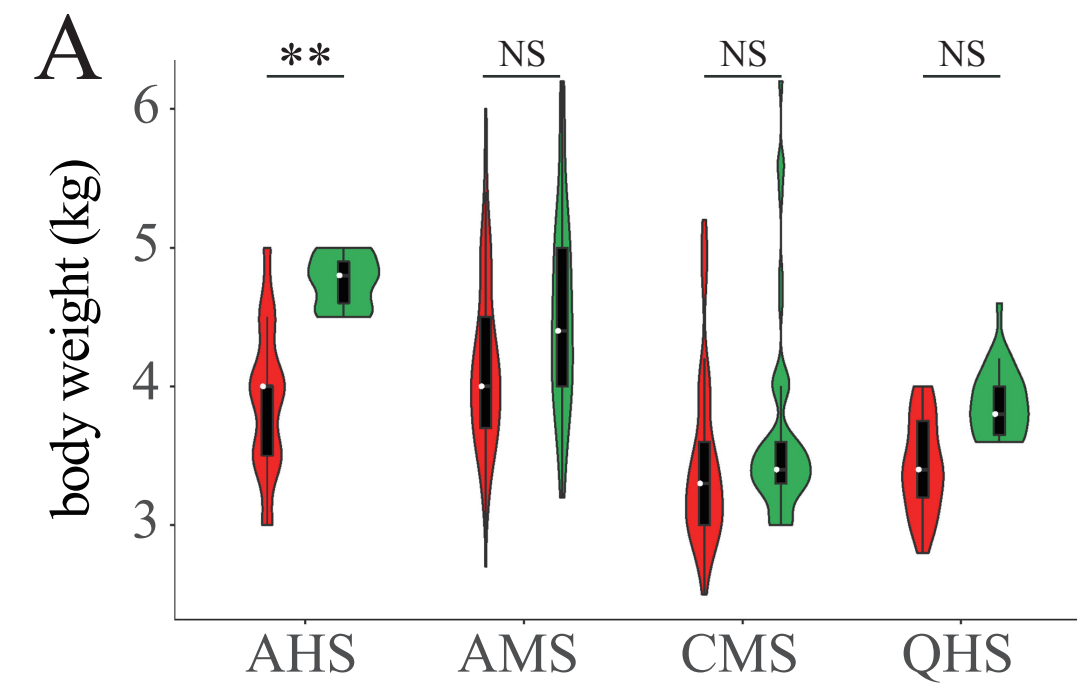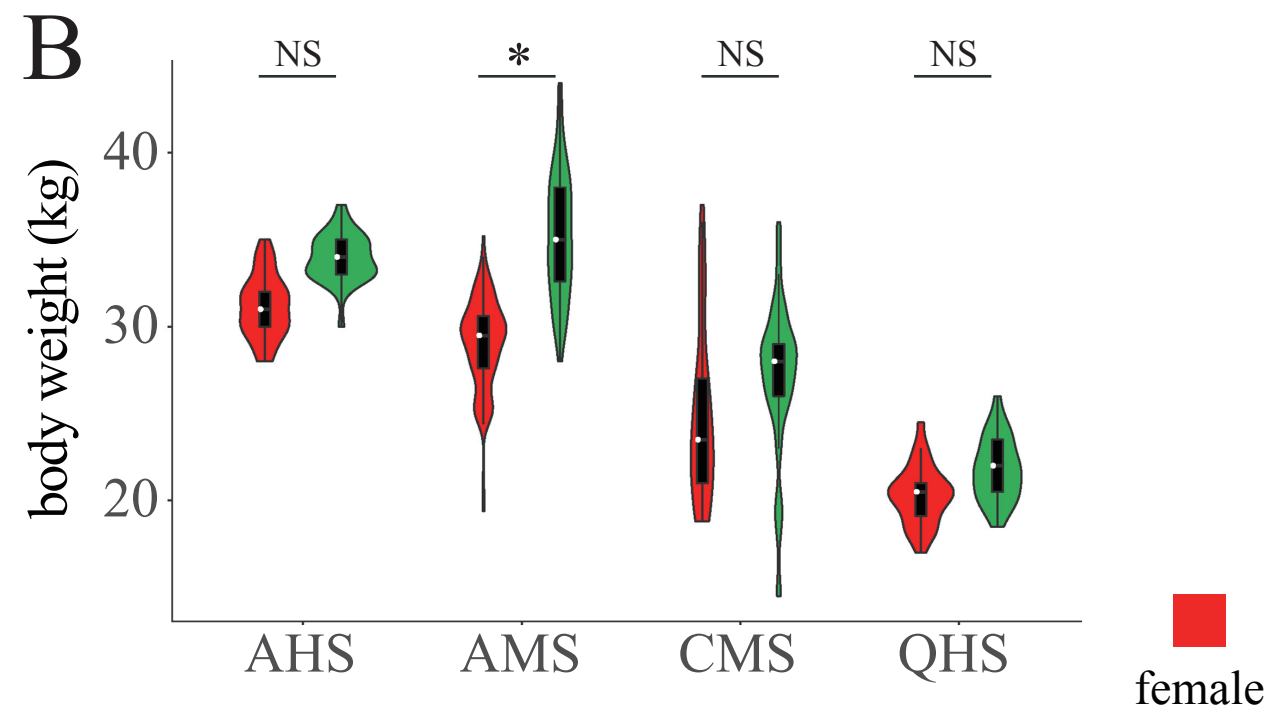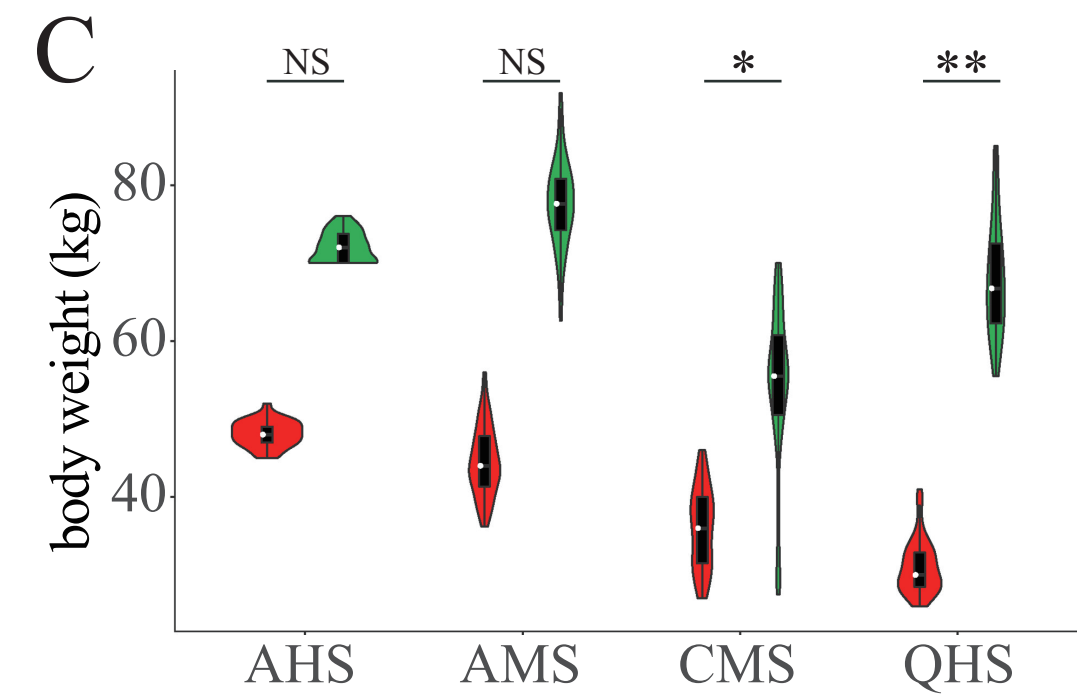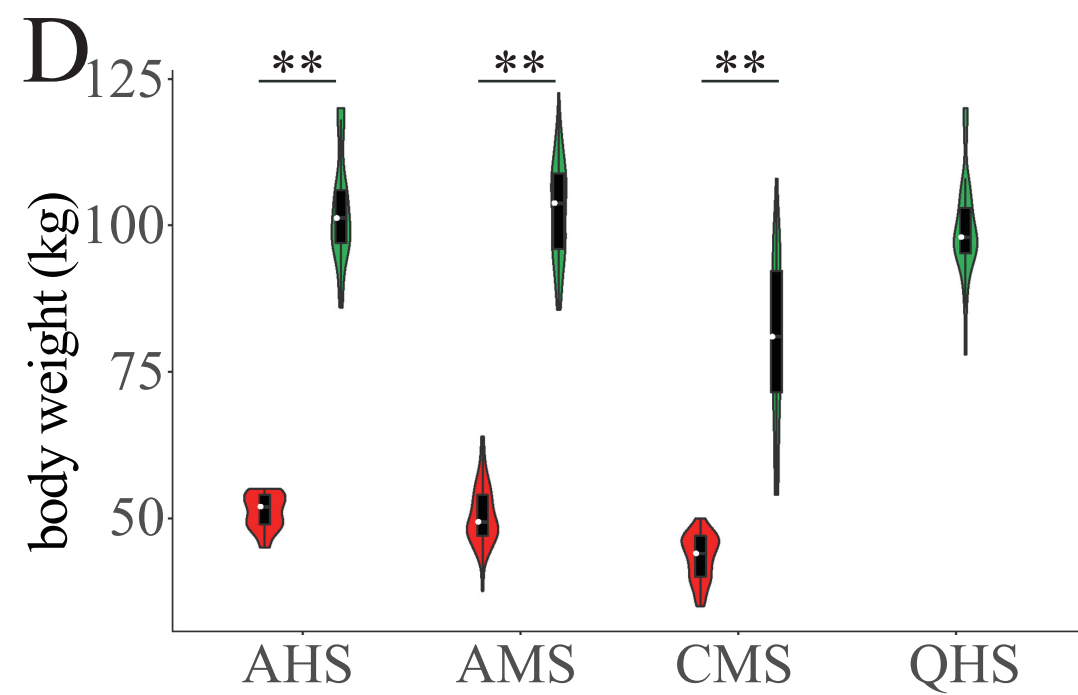

Supplement: Supplementary file 1 [file animals-10-00170-s001.zip › Supplementary/figure S1.pdf]

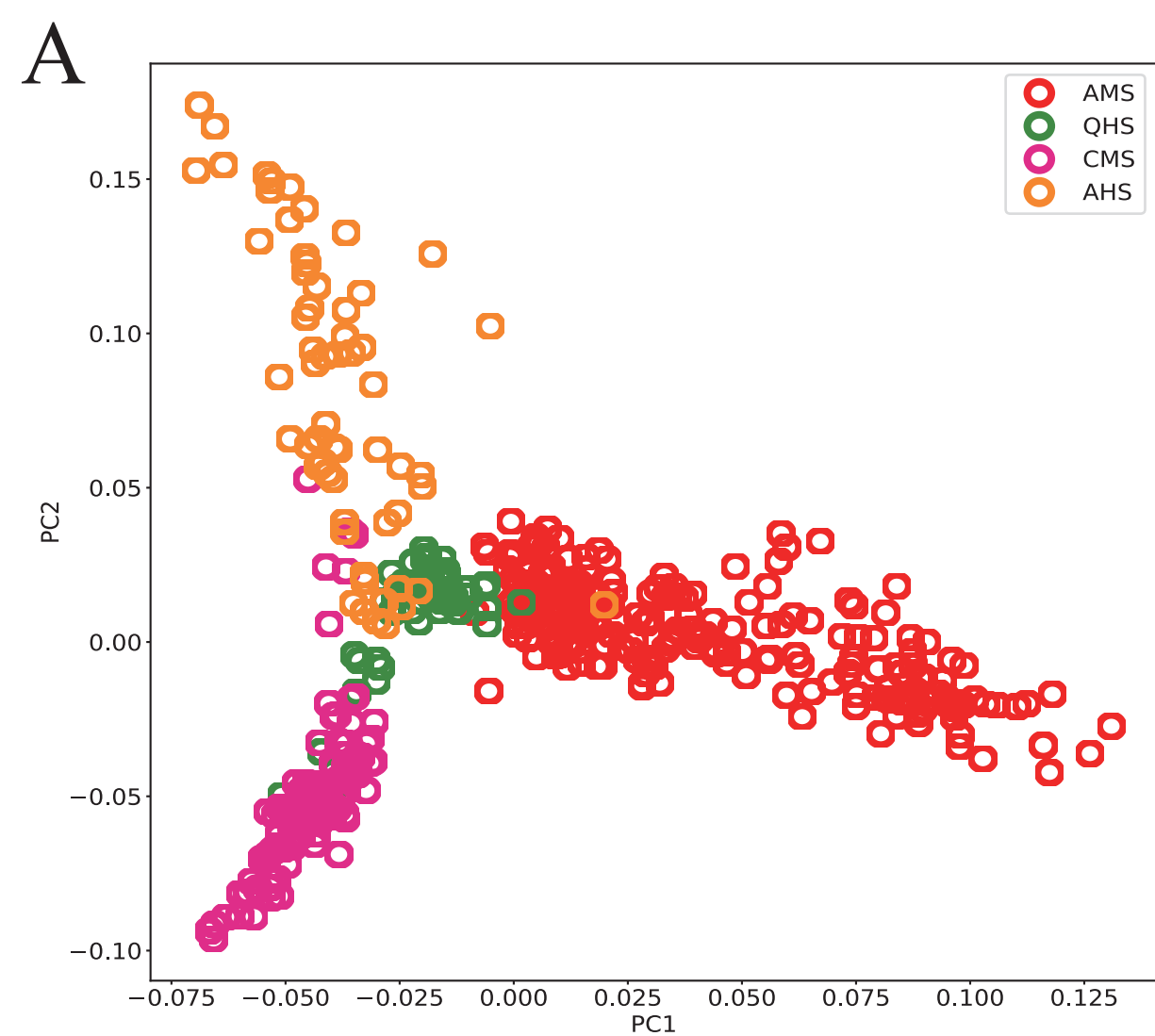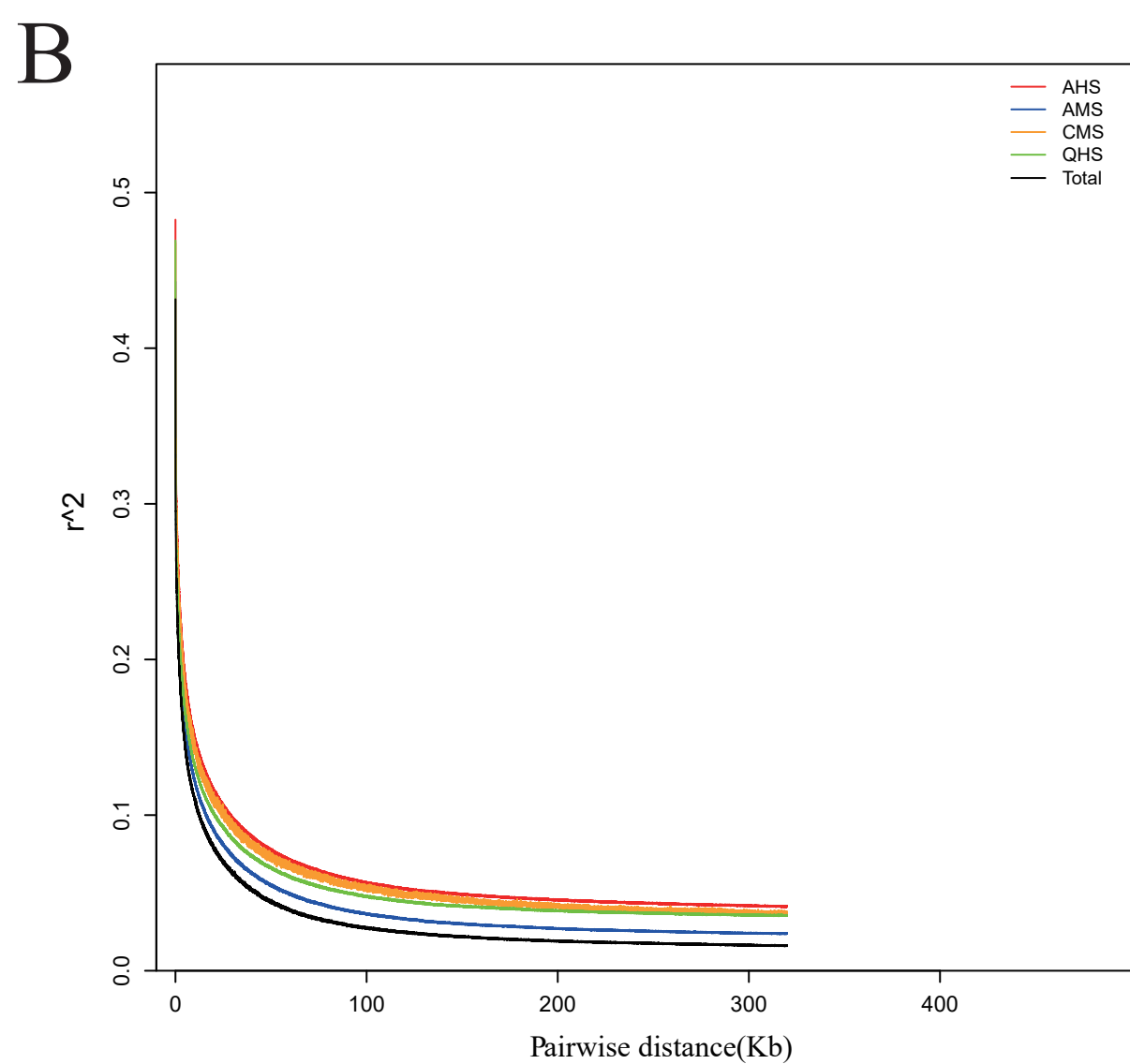

Supplement: Supplementary file 1 [file animals-10-00170-s001.zip › Supplementary/figure S2.pdf]
